# Dietary Rhythmicity and Mental Health Among Airline Personnel

**DOI:** 10.1001/jamanetworkopen.2024.22266

**Published:** 2024-07-15

**Authors:** Erliang Zhang, Huilun Li, Hangyu Han, Yuhua Wang, Shuheng Cui, Jie Zhang, Minzhi Chen, Yunfei Li, Haodong Qi, Masaki Takahashi, Mi Xiang

**Affiliations:** 1 International Peace Maternity and Child Health Hospital, School of Medicine, Shanghai Jiao Tong University, Shanghai, China; 2 Shanghai Key Laboratory of Embryo Original Diseases, Shanghai, China; 3 School of Public Health, Shanghai Jiao Tong University, Shanghai, China; 4 Department of Neurobiology, Care Sciences and Society, Karolinska Institutet, Stockholm, Sweden; 5 Malmö Institute for Studies of Migration, Diversity and Welfare, Malmö University, Malmö, Sweden; 6 Institute for Liberal Arts, Tokyo Institute of Technology, Meguro, Tokyo, Japan

## Abstract

**Importance:**

Misaligned dietary rhythmicity has been associated with metabolic diseases; however, its association with mental health remains largely unexplored.

**Objective:**

To examine the association between dietary rhythms and the mental health condition of shift workers, specifically airline crew members.

**Design, Setting, and Participants:**

This cross-sectional study analyzed data collected from the Civil Aviation Health Cohort of China, an ongoing large-scale health survey of pilots, flight attendants, and air security officers employed by major airline companies in China. Participants aged 18 to 60 years were invited through text messages to complete a web-based survey. The data collection period was December 2022 to March 2023. Statistical analysis was performed from July 24, 2023, to April 12, 2024.

**Exposure:**

Data on timing of breakfast and dinner on workdays and rest days, daily time windows for food intake, and meal and eating jet lags were collected and calculated.

**Main Outcomes and Measures:**

Anxiety and depressive symptoms were measured using the 7-item Generalized Anxiety Disorder Assessment and the 9-item Patient Health Questionnaire. Multivariate logistic regressions were performed to evaluate the associations of anxiety and depression with meal timing, eating window time, meal jet lag (ie, delayed meals), and eating jet lag (ie, delayed eating). All models were adjusted for individual socioeconomic, demographic, and lifestyle characteristics.

**Results:**

Of the 22 617 participants (median [IQR] age, 29.1 [26.3-33.7] years; 13 712 males [60.6%]), 1755 (7.8%) had anxiety and 2768 (12.2%) had depression. After controlling for confounding factors, having dinner after 8 pm on morning-shift days was associated with increased odds of anxiety (adjusted odds ratio [AOR], 1.78; 95% CI, 1.53-2.05) and depression (AOR, 2.01; 95% CI, 1.78-2.27), compared with consuming dinner before 8 pm. Similar results were observed on night-shift days and rest days. An eating window of less than 12 hours was associated with reduced odds of anxiety (AOR, 0.84; 95% CI, 0.75-0.93) and depression (AOR, 0.81; 95% CI, 0.75-0.89) on morning-shift days; the results remained significant on rest days. Delayed dinner on morning-shift days was associated with increased odds of anxiety (AOR, 1.32; 95% CI, 1.13-1.54) and depression (AOR, 1.39; 95% CI, 1.22-1.58). On night-shift days, delayed dinner was associated with higher odds of anxiety (AOR, 1.22; 95% CI, 1.06-1.39) and depression (AOR, 1.21; 95% CI, 1.08-1.36). On morning-shift days, delayed eating rhythms were associated with higher odds of depression (AOR, 1.35; 95% CI, 1.13-1.61), whereas advanced eating rhythms were associated with lower odds of anxiety (AOR, 0.78; 95% CI, 0.70-0.87).

**Conclusions and Relevance:**

This cross-sectional study found that meal timing, long eating window, and meal jet lags were associated with increased odds of depression and anxiety. These findings underscore the need for interventions and supportive policies that help mitigate the adverse implications of shift work and irregular working hours for the mental health of shift workers.

## Key Points

**Question:**

How is dietary rhythmicity associated with shift workers’ mental health?

**Findings:**

In this cross-sectional study of 22 617 airline crew members, meal timing, long eating window (over 12 hours), and delayed dinner were associated with increased odds of depression and anxiety regardless of work shift.

**Meaning:**

The findings of this study suggest the need for interventions and supportive policies that help mitigate the adverse implications of shift work and irregular working hours for mental health among airline crew members and, more broadly, among shift workers.

## Teaser

This cross-sectional study examines the association of meal timing, eating window, and dietary jet lag with odds of depression and anxiety among flight attendants, pilots, airline security staff, and other shift workers.

## Introduction

Dietary rhythmicity, emerging as a novel nutritional research field known as chrononutrition, is the concept of aligning eating patterns (eg, meal timing, daily eating window, and eating jet lag) with the body’s natural rhythms.^1,2^ Dietary rhythmicity constitutes an important aspect of nutrition that can affect human metabolism, weight, and overall well-being.^1,3^ While research suggests that irregular dietary rhythmicity is detrimental to physical health, playing a role in metabolic diseases and obesity, among others, its association with mental health has been underexamined.^4^ Irregular mealtimes and/or a prolonged eating window may disrupt microbiome circadian rhythms and may precede changes in the gut microbiota.^5^ These changes may, in turn, affect psychological health via the gut-brain axis.^5,6^

Similar to social jet lag (the difference between the midpoint of sleep time^7,8^), dietary jet lag defines the difference between the midpoint of breakfast and dinner. Both types of jet lag may interfere with the methylation of essential genes or other epigenetics and may factor in a range of metabolic health issues.^9^ The association between social jet lag and mental health conditions, such as depression, anxiety, and stress,^10,11,12,13^ is well supported by evidence. Such a relationship is potentially mediated by metabolic disease, obesity, and cardiovascular disease, among other physiological disorders.^14,15,16^ However, the link between eating jet lag and mental disorders remains largely unexplored. Since social jet lag is associated with unhealthy eating habits,^1^ eating jet lag may also be associated with mental health and hence deserves exploration.

Identifying the implications of dietary rhythmicity is challenging given that randomly assigning treatments to study participants is unethical and infeasible. In the absence of controlled experiments, shift work is often used as a measure of exposure to internal misalignment.^1^ Previous studies found that shift workers tend to have poor neurocognitive performance, more accidents and errors in and out of the workplace, and higher prevalence of mental illness.^17,18,19^ Airline crew members, a unique group of shift workers, are particularly exposed to both social and eating jet lag. The frequent shifts and rotations between day and night as well as travels across time zones among airline crews provide a unique opportunity to study the link between circadian rhythms and psychological well-being. Understanding this relationship is critical to developing interventions for mitigating airline crew members’ stress and fatigue risks from shift work and jet lag. Such interventions, in a broader context, are critical to ensuring the safety of air travel passengers (over 8 billion annually). Thus, this study aimed to investigate the association between the mental health condition and meal timing, eating window, and dietary jet lag of shift workers, specifically airline crew members.

## Methods

### Study Design and Participants

This cross-sectional study is a secondary analysis of deidentified data collected by the Civil Aviation Health Cohort of China (CAHCC). The CAHCC is an ongoing large-scale health surveillance cohort aiming to promote physical and mental health among pilots, flight attendants, and aviation security staff in China. The present study analyzed data collected in the first study wave, which was conducted from December 2022 to March 2023 and involved 10 Chinese airline companies. The collaborating airlines were at varying capacities, ranging from large (more than 1000 staff) to small (more than 100 staff). Both passenger and cargo airlines with domestic and international routes were included. More details about the CAHCC are provided in the eMethods in Supplement 1. The Ethics Committee of Civil Aviation Shanghai Hospital approved the CAHCC. All airline companies consented to collaborate, and all airline crew members provided online consent to participate. Ethics approval and consent extended to this secondary analysis. We followed the Strengthening the Reporting of Observational Studies in Epidemiology (STROBE) reporting guideline.

A web-based survey was distributed to the 10 collaborating airline companies. Text messages containing a full description of the research and an access link to the questionnaire were sent via the Wenjuanxing platform to the airlines’ employees aged 18 to 60 years.

### Measures

#### Meal Timing and Daily Eating Window

Work shifts for airline crew members are irregular. During a typical week, they may operate early flights on some days (morning-shift days) and late flights on other days (night-shift days). To capture the variability of breakfast and dinner times during different work shifts, we relied on this survey question: What time do you usually have breakfast and dinner when working morning shifts (takeoff before 09:00), night shifts (landing after 22:00), and on rest days, respectively? Daily eating window time is typically defined as the interval between the first and last caloric intake each day.^20^ In this study, we used the interval between the first and the last meal of the day (ie, between breakfast and dinner). Breakfast times were grouped as before 8 am, between 8 and 9 am, and after 9 am, and dinner times were grouped as before 8 pm and after 8 pm. These time thresholds were chosen because they were considered to be associated with metabolism.^21^ A daily eating window of 12 hours was widely used in time-restricted eating studies.^2,22^ Following the literature, we grouped eating windows as over 12 hours and 12 hours or less.

#### Meal Jet Lag and Eating Jet Lag

Dietary jet lag, defined as the variability of eating time, was estimated using the method for calculating social jet lag.^10^ For meal jet lag, we took the difference between mealtime on workdays (morning-shift days and night-shift days) and on rest days. Breakfasts and dinners were calculated separately.

For eating jet lag, we defined the eating midpoint as the intermediate time between the first and the last meal. Similar to the method of estimating the sleep midpoint,^11^ we then took the difference between eating midpoint on weekdays (early-shift days and late-shift days) and on rest days.^1^ Following the literature on social jet lag,^10^ we grouped eating jet lag as 1 hour or more or less than 1 hour.

Finally, we defined meal jet lag and eating jet lag as delayed, advanced, or maintained according to the timing of each meal on weekdays. Specifically, if the value was less than 1 hour, mealtime was considered to be advanced; if the value was more than 1 hour, mealtime was considered to be delayed; and if the value was within 1 hour, mealtime was considered to be maintained.^20^

### Mental Health Outcomes

The 7-item Generalized Anxiety Disorder (GAD-7) Assessment was used to assess anxiety.^23^ Depression was measured by the 9-item Patient Health Questionnaire (PHQ-9).^24^ The total scores ranged from 0 to 21 for the GAD-7 and from 0 to 27 for the PHQ-9, with higher scores indicating higher levels of anxiety or depression. Participants were categorized as having anxiety or depression if they scored 10 or higher on GAD-7 or PHQ-9.^25,26^ The Chinese versions of these 2 scales were validated previously.^27^

Participants also reported their age, sex, educational level, marital status, weight and height, occupation, personal annual income, total flight participation hours, ratio of the number of morning shifts to the number of night shifts per week, daily sleep duration, social jet lag, physical activity level, tobacco use, and alcohol use. All measurements are described in the eMethods in Supplement 1.

### Statistical Analysis

Data analysis was conducted from July 24, 2023, to April 12, 2024, using SPSS version 25 (IBM) and R version 4.3.2 (R Project for Statistical Computing). Descriptive characteristics of airline crew members were presented in medians and IQRs for continuous variables and frequencies and percentages for categorical variables. The Mann-Whitney test was used for continuous variables, and the χ^2^ test was used for categorical variables in comparing characteristics by mental health status. A series of multivariate logistic regressions were performed to examine the association of anxiety and depression with meal timing, eating window time, meal jet lag, and eating jet lag. All models were adjusted for age, sex, educational level, marital status, body mass index (calculated as weight in kilograms divided by height in meters squared), occupation, personal annual income, total flight participation hours, ratio of the number of morning shifts to the number of night shifts per week, daily sleep duration, social jet lag, physical activity level, tobacco use, and alcohol use.

Two-tailed *P* < .05 was considered to be statistically significant after correction for false discovery rate using the Benjamini-Hochberg method. Sex-specific analyses were also performed.

## Results

A total of 24 760 airline crew members from 10 airline companies were invited to complete online questionnaires. Of these employees, 22 617 completed the first survey, for a 91.3% response rate. This study population consisted of 8905 males (39.4%) and 13 712 females (60.6%), with a median (IQR) age of 29.1 (26.3-33.7) years, of whom 34.9% reported being pilots, 15.5% being security officers, and 49.6% being flight attendants. Overall, 1755 participants (7.8%) had a score of 10 or higher on the GAD-7, and 2768 participants (12.2%) had a score of 10 or higher on the PHQ-9 (Table).

**Table.** Demographic Characteristics of Study Participants

Characteristic	Participants, No. (%)			*P* value^b^	Participants, No. (%)		*P* value^b^
	Overall (n = 22 617)	Without anxiety (n = 20 862)	With anxiety (n = 1755)^a^		Without depression (n = 19 849)	With depression (n = 2768)^a^	
Age, median (IQR), y	29.1 (26.3-33.7)	29.2 (26.3-33.8)	28.7 (26.1-33.0)	.001	29.2 (26.3-33.8)	28.7 (26.1-33.0)	<.001
Sex							
Male	8905 (39.4)	8038 (38.5)	867 (49.4)	<.001	7498 (37.8)	1407 (50.8)	<.001
Female	13 712 (60.6)	12 824 (61.5)	888 (50.6)		12 351 (62.2)	1361 (49.2)	
Marital status							
Single	7652 (33.8)	6974 (33.4)	678 (38.6)	<.001	6553 (33.0)	1099 (39.7)	<.001
With long-term partner	4194 (18.5)	3838 (18.4)	356 (20.3)		3644 (18.4)	550 (19.9)	
Married	10 283 (45.5)	9617 (46.1)	666 (37.9)		9245 (46.6)	1038 (37.5)	
Divorced or widowed	488 (2.2)	433 (2.1)	55 (3.1)		407 (2.1)	81 (2.9)	
Educational level							
≤Junior college	8454 (37.4)	7717 (37.0)	737 (42.0)	<.001	7258 (36.6)	1196 (43.2)	<.001
Bachelor’s degree	13 859 (61.3)	12 863 (61.7)	996 (56.8)		12 324 (62.1)	1535 (55.5)	
≥Master’s degree	304 (1.3)	282 (1.4)	22 (1.3)		267 (1.3)	37 (1.3)	
BMI							
<18.5	3999 (17.7)	3603 (17.3)	396 (22.6)	<.001	3376 (17.0)	623 (22.5)	<.001
18.5-23.9	12 072 (53.4)	11 182 (53.6)	890 (50.7)		10 652 (53.7)	1420 (51.3)	
≥24.0	6546 (28.9)	6077 (29.1)	469 (26.7)		5821 (29.3)	725 (26.2)	
Occupation							
Flight attendant	11 224 (49.6)	10 101 (48.4)	1123 (64.0)	<.001	9417 (47.4)	1807 (65.3)	<.001
Security officer	3509 (15.5)	3187 (15.3)	322 (18.3)		3022 (15.2)	487 (17.6)	
Pilot	7884 (34.9)	7574 (36.3)	310 (17.7)		7410 (37.3)	474 (17.1)	
Personal annual income, ¥							
<50 000	4535 (20.1)	4032 (19.3)	503 (28.7)	<.001	3831 (19.3)	704 (25.4)	<.001
50 000-150 000	11 918 (52.7)	10 909 (52.3)	1009 (57.5)		10 247 (51.6)	1671 (60.4)	
>150 000	6164 (27.3)	5921 (28.4)	243 (13.8)		5771 (29.1)	393 (14.2)	
Total flight participation hours, median (IQR)	3500 (1500-7550)	3500 (1500-7600)	3600 (1800-7000)	.05	3500 (1500-7600)	1800 (3800-7400)	<.001
Ratio of number of morning shifts to number of night shifts per week, median (IQR)	1.00 (1.00-1.48)	1.00 (1.00-1.39)	1.00 (1.00-1.48)	<.001	1.00 (1.00-1.32)	1.00 (1.00-1.48)	<.001
Physical activity, min/wk							
<150	8854 (39.1)	7964 (38.2)	890 (50.7)	<.001	7502 (37.8)	1352 (48.8)	<.001
≥150	13 763 (60.9)	12 898 (61.8)	865 (49.3)		12 347 (62.2)	1416 (51.2)	
Daily sleep duration, h/d							
<7	5283 (23.4)	4495 (21.5)	788 (44.9)	<.001	4132 (20.8)	1151 (41.6)	<.001
≥7	17 334 (76.6)	16 367 (78.5)	967 (55.1)		15 717 (79.2)	1617 (58.4)	
Social jet lag, h							
<1	6871 (30.4)	6440 (30.9)	431 (24.6)	<.001	6104 (30.8)	767 (27.7)	.001
≥1	15 746 (69.6)	14 422 (69.1)	1324 (75.4)		13 745 (69.2)	2001 (72.3)	
Tobacco use							
Never	16 231 (71.8)	15 043 (72.1)	1188 (67.7)	<.001	14 357 (72.3)	1874 (67.7)	<.001
Former	1707 (7.5)	1550 (7.4)	157 (8.9)		1464 (7.4)	243 (8.8)	
Current	4679 (20.7)	4269 (20.5)	410 (23.4)		4028 (20.3)	651 (23.5)	
Alcohol use, d/mo							
0	14715 (65.1)	13 705 (65.7)	1010 (57.5)	<.001	13 134 (66.2)	1581 (57.1)	<.001
1	2720 (12.0)	2484 (11.9)	236 (13.4)		2337 (11.8)	383 (13.8)	
≥2	5182 (22.9)	4673 (22.4)	509 (29.0)		4378 (22.1)	804 (29.0)	
Breakfast timing							
Morning-shift days							
Before 8 AM	18 382 (81.3)	17 051 (81.7)	1331 (75.8)	<.001	16 304 (82.1)	2078 (75.1)	<.001
Between 8 AM and 9 AM	1708 (7.6)	1605 (7.7)	103 (5.9)		1530 (7.7)	178 (6.4)	
After 9 AM	2527 (11.2)	2206 (10.6)	321 (18.3)		2015 (10.2)	512 (18.5)	
Night-shift days							
Before 8 AM	8451 (37.4)	7671 (36.8)	780 (44.4)	<.001	7336 (37.0)	1115 (40.3)	<.001
Between 8 AM and 9 AM	4157 (18.4)	3981 (19.1)	176 (10.0)		3873 (19.5)	284 (10.3)	
After 9 AM	10 009 (44.3)	9210 (44.1)	799 (45.5)		8640 (43.5)	1369 (49.5)	
Rest days							
Before 8 AM	7732 (34.2)	6994 (33.5)	738 (42.1)	<.001	6723 (33.9)	1009 (36.5)	<.001
Between 8 AM and 9 AM	5052 (22.3)	4828 (23.1)	224 (12.8)		4690 (23.6)	362 (13.1)	
After 9 AM	9833 (43.5)	9040 (43.3)	793 (45.2)		8436 (42.5)	1397 (50.5)	
Dinner timing							
Morning-shift days							
Before and 8 PM	20905 (92.4)	19 442 (93.2)	1463 (83.4)	<.001	18 605 (93.7)	2300 (83.1)	<.001
After 8 PM	1712 (7.6)	1420 (6.8)	292 (16.6)		1244 (6.3)	468 (16.9)	
Night-shift days							
Before and 8 PM	19 700 (87.1)	18 336 (87.9)	1364 (77.7)	<.001	17 548 (88.4)	2152 (77.7)	<.001
After 8 PM	2917 (12.9)	2526 (12.1)	391 (22.3)		2301 (11.6)	616 (22.3)	
Rest days							
Before and 8 PM	21 305 (94.2)	19 748 (94.7)	1557 (88.7)	<.001	18 867 (95.1)	2438 (88.1)	<.001
After 8 PM	1312 (5.8)	1114 (5.3)	198 (11.3)		982 (4.9)	330 (11.9)	
Eating window time, h							
Morning-shift days							
>12	7322 (32.4)	6637 (31.8)	685 (39.0)	<.001	6232 (31.4)	1090 (39.4)	<.001
≤12	15 295 (67.6)	14 225 (68.2)	1070 (61.0)		13 617 (68.6)	1678 (60.6)	
Night-shift days							
>12	1315 (5.8)	1172 (5.6)	143 (8.1)	<.001	1091 (5.5)	224 (8.1)	<.001
≤12	21 302 (94.2)	19 690 (94.4)	1612 (91.9)		18 758 (94.5)	2544 (91.9)	
Rest days							
>12	511 (2.3)	445 (2.1)	66 (3.8)	<.001	414 (2.1)	97 (3.5)	<.001
≤12	22 106 (97.7)	20 417 (97.9)	1689 (96.2)		19435 (97.9)	2671 (96.5)	
Breakfast jet lag							
Morning-shift days							
Maintained	8722 (38.6)	7937 (38.0)	785 (44.7)	<.001	7601 (38.3)	1121 (40.5)	<.001
Advanced	12 961 (57.3)	12 099 (58.0)	862 (49.1)		11 476 (57.8)	1485 (53.6)	
Delayed	934 (4.1)	826 (4.0)	108 (6.2)		772 (3.9)	162 (5.9)	
Night-shift days							
Maintained	18 518 (81.9)	17 096 (81.9)	1422 (81.0)	.60	16 247 (81.9)	2271 (82.0)	.22
Advanced	2260 (10.0)	2079 (10.0)	181 (10.3)		1968 (9.9)	292 (10.5)	
Delayed	1839 (8.1)	1687 (8.1)	152 (8.7)		1634 (8.2)	205 (7.4)	
Dinner jet lag							
Morning-shift days							
Maintained	18 951 (83.8)	17 586 (84.3)	1365 (77.8)	<.001	16 818 (84.7)	2133 (77.1)	<.001
Advanced	1843 (8.1)	1695 (8.1)	148 (8.4)		1581 (8.0)	262 (9.5)	
Delayed	1823 (8.1)	1581 (7.6)	242 (13.8)		1450 (7.3)	373 (13.5)	
Night-shift days							
Maintained	18 714 (82.7)	17 361 (83.2)	1353 (77.1)	<.001	16 596 (83.6)	2118 (76.5)	<.001
Advanced	1004 (4.4)	912 (4.4)	92 (5.2)		838 (4.2)	166 (6.0)	
Delayed	2899 (12.8)	2589 (12.4)	310 (17.7)		2415 (12.2)	484 (17.5)	
Eating jet lag							
Morning-shift days							
Maintained	12 066 (53.3)	11 052 (53.0)	1014 (57.8)	<.001	10 591 (53.4)	1475 (53.3)	<.001
Advanced	9684 (42.8)	9063 (43.4)	621 (35.4)		8575 (43.2)	1109 (40.1)	
Delayed	867 (3.8)	747 (3.6)	120 (6.8)		683 (3.4)	184 (6.6)	
Night-shift days							
Maintained	18 544 (82.0)	17 174 (82.3)	1370 (78.1)	<.001	16 379 (82.5)	2165 (78.2)	<.001
Advanced	1549 (6.8)	1419 (6.8)	130 (7.4)		1323 (6.7)	226 (8.2)	
Delayed	2524 (11.2)	2269 (10.9)	255 (14.5)		2147 (10.8)	377 (13.6)	

Abbreviations: BMI, body mass index (calculated as weight in kilograms divided by height in meters squared); ¥, Chinese yuan renminbi (to convert to US dollar, multiply by 7.18).

^a^ Anxiety and depression were defined as a score of 10 or higher on the 7-item Generalized Anxiety Disorder Assessment (score range: 0-21, with higher scores indicating higher levels of anxiety) and the 9-item Patient Health Questionnaire (score range: 0-27, with higher scores indicating higher levels of depression), respectively.

^b^ *P* values were calculated using the Mann-Whitney test for continuous variables or the χ^2^ test for categorical variable.

Meal timing tends to vary noticeably across work shifts. When working morning shifts, 11.2% of participants reported having late breakfast (after 9 am), and 7.6% had late dinner (after 8 pm). However, on night-shift days, the corresponding proportions increased to 44.3% for breakfast and 12.9% for dinner. These patterns indicate that mealtimes tended to vary depending on the flight shifts. Regarding breakfast jet lag, most airline crew members on morning-shift days had early breakfast (57.3% advanced) compared with rest days. In contrast, breakfast time was maintained on night shifts. For dinner jet lag, more than 80% of participants maintained dinner time on rest days regardless of their shifts. Nearly all participants (97.7%) reported eating all meals within 12 hours on a rest day. However, the proportions decreased to 67.6% when crews were on morning shifts and to 94.2% when on night shifts. For overall eating jet lag (ie, change in the midpoint between breakfast and dinner), most airline crew members maintained their eating rhythms similar to those on rest days. No eating jet lag was reported by 53.3% of participants when working on morning shifts and by 82.0% of participants when working on night shifts (Table).

**Figure 1.**
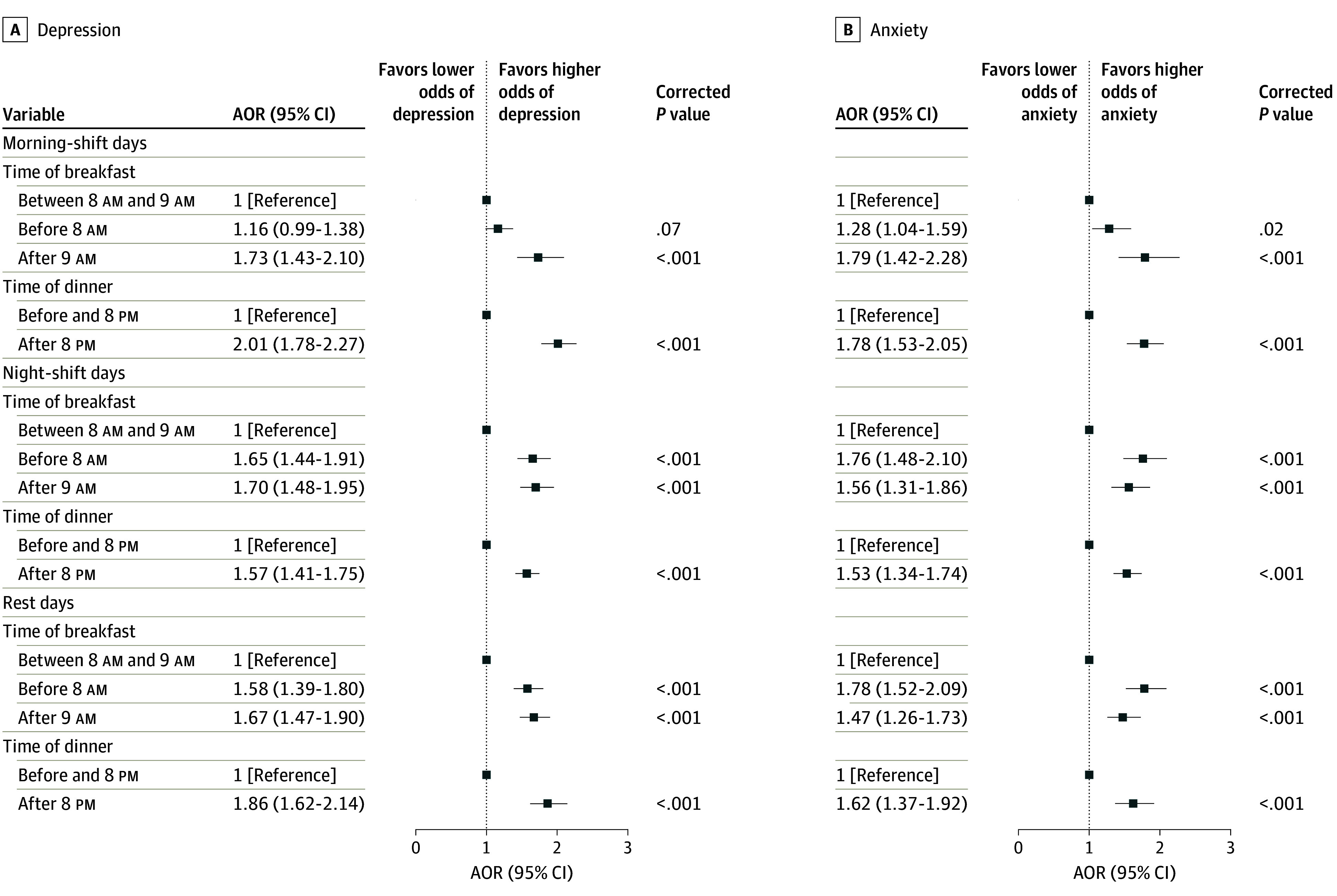
Associations Between Meal Timing and Mental Health Outcomes The odds ratios were adjusted for age, sex, educational level, marital status, body mass index (calculated as weight in kilograms divided by height in meters squared), occupation, personal annual income, total flight participation hours, ratio of the number of morning shifts to the number of night shifts per week, daily sleep duration, social jet lag, physical activity level, tobacco use, and alcohol use. All *P* values are presented after Benjamini-Hochberg multiple testing adjustments. AOR indicates adjusted odds ratio.

Figure 1 depicts the associations between meal timing and mental health outcomes. Compared with the reference category (breakfast between 8 am and 9 am), having an early breakfast was associated with depression (adjusted odds ratio [AOR], 1.65 [95% CI, 1.44-1.91] on night-shift days; AOR, 1.58 [95% CI, 1.39-1.80] on rest days) and with anxiety (AOR, 1.28 [95% CI, 1.04-1.59] on morning-shift days; AOR, 1.76 [95% CI, 1.48-2.10] on night-shift days; AOR, 1.78 [95% CI, 1.52-2.09] on rest days). Moreover, late breakfast (after 9 am) was also associated with depression (AOR, 1.73 [95% CI, 1.43-2.10] on morning-shift days; AOR, 1.70 [95% CI, 1.48-1.95] on night-shift days; AOR, 1.67 [95% CI, 1.47-1.90] on rest days) and with anxiety (AOR, 1.79 [95% CI, 1.42-2.28] on morning-shift days; AOR, 1.56 [95% CI, 1.31-1.86] on night-shift days; AOR, 1.47 [95% CI, 1.26-1.73] on rest days).

Compared with the reference group (before 8 pm), airline crew members who had late dinner had higher odds of depression (AOR, 2.01 [95% CI, 1.78-2.27] on morning-shift days; AOR, 1.57 [95% CI, 1.41-1.75] on night-shift days; AOR, 1.86 [95% CI, 1.62-2.14] on rest days). A similar pattern was observed for anxiety, with a slight decrease in the estimated odds (AOR, 1.78 [95% CI, 1.53-2.05] on morning-shift days; AOR, 1.53 [95% CI, 1.34-1.74] on night-shift days; AOR, 1.62 [95% CI, 1.37-1.92] on rest days).

**Figure 2.**
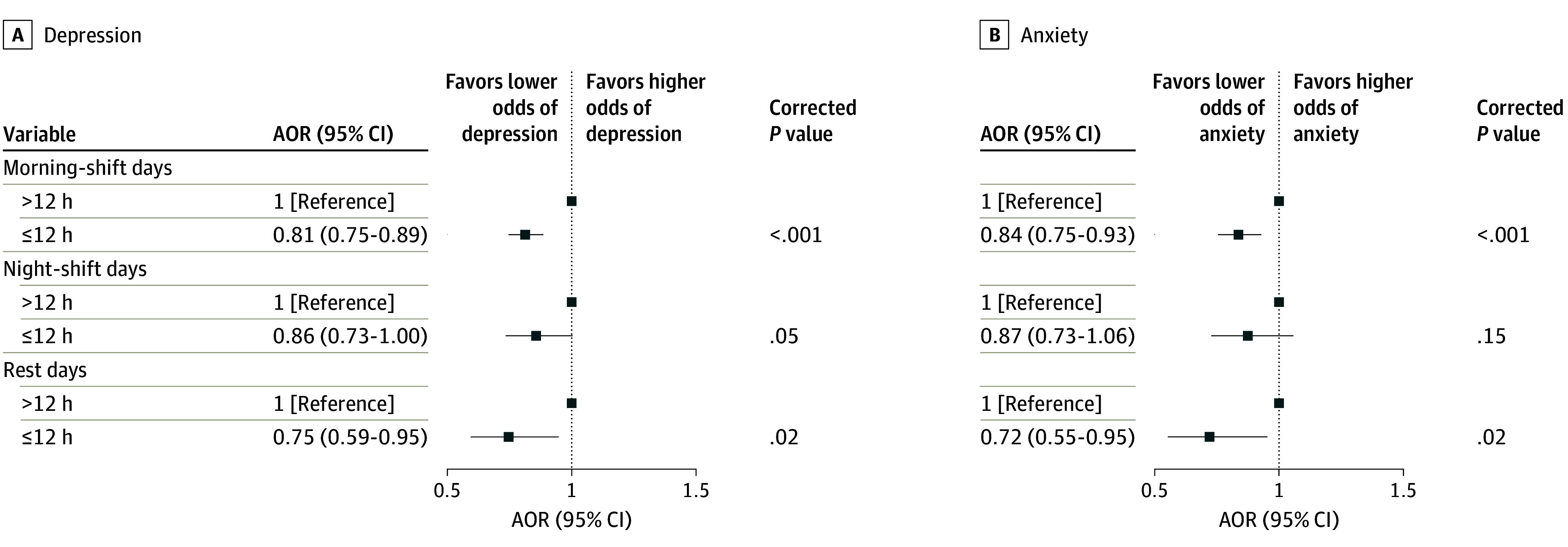
Associations Between Eating Window and Mental Health Outcomes The odds ratios were adjusted for age, sex, educational level, marital status, body mass index (calculated as weight in kilograms divided by height in meters squared), occupation, personal annual income, total flight participation hours, ratio of the number of morning shifts to the number of night shifts per week, daily sleep duration, social jet lag, physical activity level, tobacco use, and alcohol use. All *P* values are presented after Benjamini-Hochberg multiple testing adjustments. AOR indicates adjusted odds ratio.

Figure 2 illustrates the associations between eating window and mental health outcomes. Participants who reported having all meals within 12 hours showed a lower occurrence of depression (AOR, 0.81 [95% CI, 0.75-0.89] on morning-shift days; AOR, 0.75 [95% CI, 0.59-0.95] on rest days). They also reported lower odds of anxiety (AOR, 0.84 [95% CI, 0.75-0.93] on morning-shift days; AOR, 0.72 [95% CI, 0.55-0.95] on rest days).

**Figure 3.**
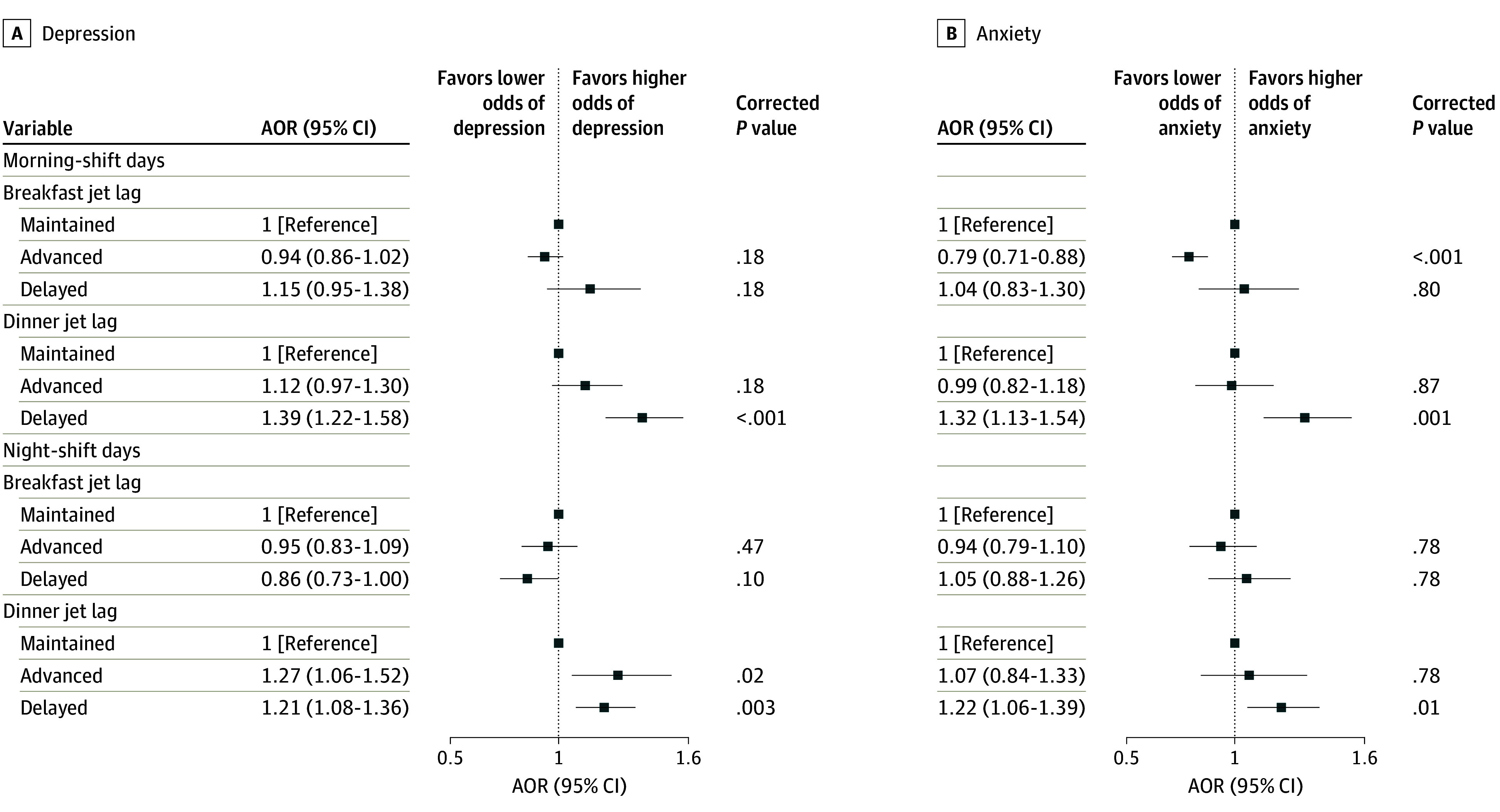
Associations Between Meal Jet Lags and Mental Health Outcomes The odds ratios were adjusted for age, sex, educational level, marital status, body mass index (calculated as weight in kilograms divided by height in meters squared), occupation, personal annual income, total flight participation hours, ratio of the number of morning shifts to the number of night shifts per week, daily sleep duration, social jet lag, physical activity level, tobacco use, and alcohol use. All *P* values are presented after Benjamini-Hochberg multiple testing adjustments. AOR indicates adjusted odds ratio.

The associations between meal jet lags and mental health outcomes are shown in Figure 3. Airline crew members who had breakfasts ahead of time had lower odds of anxiety (AOR, 0.79; 95% CI, 0.71-0.88). Those who had delayed dinners displayed an increased odds of depression (AOR, 1.39 [95% CI, 1.22-1.58] on morning-shift days; AOR, 1.21 [95% CI, 1.08-1.36] on night-shift days) and anxiety (AOR, 1.32 [95% CI, 1.13-1.54] on morning-shift days; AOR, 1.22 [95% CI, 1.06-1.39] on night-shift days).

**Figure 4.**
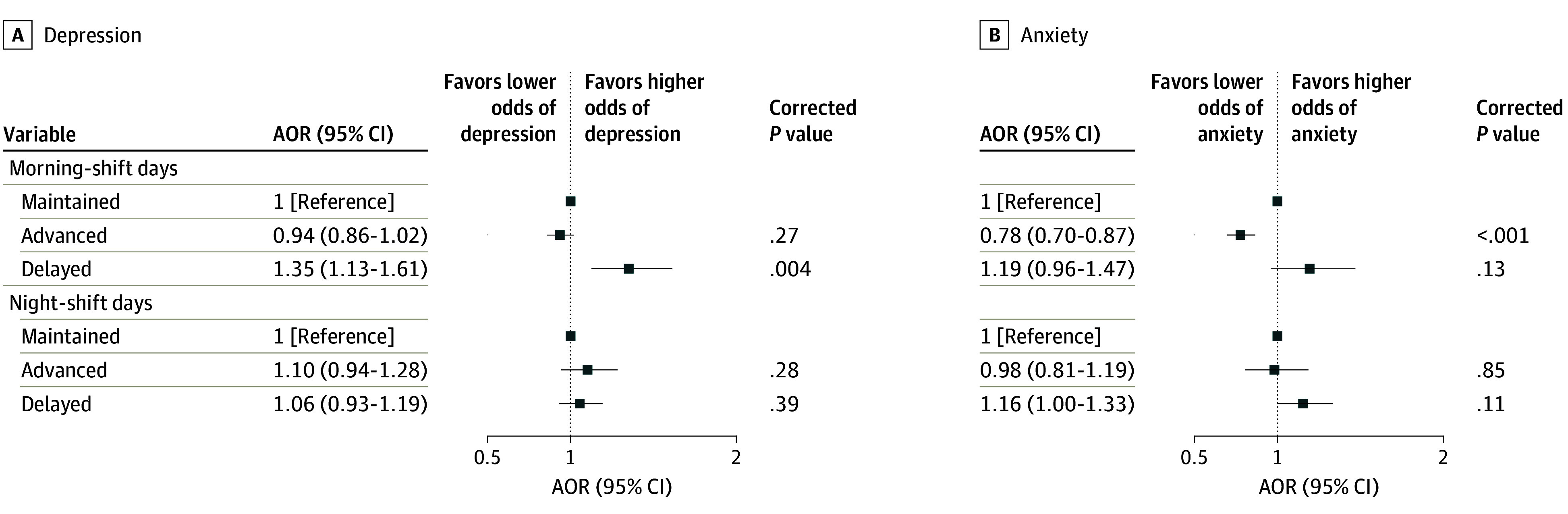
Associations Between Eating Jet Lags and Mental Health Outcomes The odds ratios were adjusted for age, sex, educational level, marital status, body mass index (calculated as weight in kilograms divided by height in meters squared), occupation, personal annual income, total flight participation hours, ratio of the number of morning shifts to the number of night shifts per week, daily sleep duration, social jet lag, physical activity level, tobacco use, and alcohol use. All *P* values are presented after Benjamini-Hochberg multiple testing adjustments. AOR indicates adjusted odds ratio.

Figure 4 shows the associations between eating jet lags (the changes in midpoints between breakfasts and dinners) and mental health outcomes. There was no association on night-shift days. In the case of morning shifts, associations were observed. Delayed eating rhythms were associated with higher odds of depression (AOR, 1.35; 95% CI, 1.13-1.61) compared with the reference. Conversely, advanced eating jet lag was associated with lower odds of anxiety (AOR, 0.78; 95% CI, 0.70-0.87).

To further clarify the differences by sex, we conducted sex-stratified analyses. We obtained similar results for both males and females (eFigures 1 to eFigure 8 in Supplement 1).

## Discussion

This study examined the association between dietary rhythmicity and mental health. By examining airline crew members working for major Chinese airlines, we found that the dietary rhythms of individuals in this profession varied depending on the time of flight operations (early morning or late night). These irregular eating rhythms were associated with higher odds of anxiety and depression. To our knowledge, the CAHCC is the largest population-based study of the relationship between dietary rhythmicity and psychological well-being in airline crew members. Although this study targeted a specific occupation group, the large sample size allowed us to conduct a comprehensive analysis of the relationship between mental health and different aspects of dietary rhythms, including variability of mealtime, eating windows, and dietary jet lags. It also allowed us to obtain reliable estimates by controlling for a variety of individual characteristics (socioeconomic, demographic, and lifestyle) that might confound these associations.

Shift workers, such as nurses, medical interns, radiologic technicians, police officers, railway workers, and industrial workers, are subject to mental health conditions.^28,29,30^ However, the magnitude of the association of shift work with mental health may vary depending on job characteristics and the degree of irregularities in work schedules. For example, a survey conducted in the US revealed that fatigue and depression levels among flight attendants were approximately twice as high as the levels in the general population.^31^ Moreover, at the intensive margin, airline crews’ mental well-being was found to be associated with the length of flight routes and the number of delays encountered as well as the number of workdays and the time spent commuting to work.^32^ More research is needed to reveal the unique stressors and challenges faced by airline crew members and to develop intervention programs accordingly. The findings may also have social benefits since the health of aviation personnel is essential for ensuring public safety.

The present study innovatively centered on meal timing, eating time windows, and meal jet lags. The results indicated that the dietary rhythms and eating jet lags from the shift work patterns of airline crew members have an inverse association with mental health. An eating window of less than 12 hours may be associated with reduced severity of anxiety or depression. From the perspective of eating jet lag, the fluctuation of meal timing was also associated with the odds of anxiety and depression in shift workers. Advanced breakfast was associated with reduced odds of anxiety. These findings established the association between dietary rhythms and mental health.

Several potential mechanisms underlying such relationships are discussed in previous literature. Untimely and irregular eating times may affect the circadian rhythm of the gut microbiota. The circadian rhythm of the peripheral clock is regulated by food intake,^6^ and the subsequent increase in insulin secretion after food intake induces the regulatory pathway of clock genes, such as *PI3/AKT*.^33^ Irregular mealtimes are associated with metabolic shifts, such as changes in leptin or growth hormone–releasing peptide function, which in turn are associated with impaired mental health.^34^ Additionally, food intake may modulate the gastrointestinal system, including the gut microbiota and enteroendocrine cells, which send signals to the central nervous system via the gut-brain axis.^5^ Time-restricted eating, as 1 indicator of meal timing, plays a role in improved neurogenesis and synaptic plasticity, thereby delaying cognitive decline and neurodegeneration. Several cellular mechanisms are thought to contribute to the potential benefits of time-restricted feeding for the nervous system, including enhanced neurotrophic factor signaling, reduced accumulation of oxidative damage molecules, and reduced neuroinflammation.^3^ These mechanisms might explain why we observed in this analysis increased odds of anxiety and depression when the eating window was longer than 12 hours. Moreover, similar to social jet lag,^14,15,35^ dietary jet lag may alter the methylation of 5 crucial genes and may be associated with a range of metabolic health issues. It may also be a factor in impaired mental health by reducing neuronal complexity, cognitive flexibility, and executive function.^6^ Such mechanisms may underlie the associations between eating jet lag and mental health outcomes examined in this analysis.

Through rigorous training and resilience building, airline crews are presumed to be more capable of coping better with stress and managing emergencies than average workers. This selectivity implies that the estimates obtained from this analysis are likely to be at the lower bounds. Hence, for a typical shift worker, the psychological consequences of misaligned circadian rhythmicity could be more severe. To gain a more comprehensive view of such consequences, studies that compare populations with different job characteristics, as well as across different contexts and geographies, could be beneficial. Such a comparative analysis may be the focus of future research.

### Limitations

Several limitations of this study are worth noting. First, due to the cross-sectional nature of the CAHCC data, we could not identify any causal associations. Despite the models adjusting for a large set of confounding factors, unobserved heterogeneity may still pertain to the model residuals; should they correlate with meal timing and eating jet lag, the parameter estimates do not reflect causal associations. Second, given their rigorous training in emergency procedures and crisis management, among others, airline crew members are typically more resilient than average employees. Thus, the associations between dietary rhythm and mental health we found in this population are likely to be smaller and less significant, compared with the associations found in other shift workers.^29^ Third, although the measures of key variables (eg, mealtimes and mental health measures) were constructed using validated methods, they might still be subject to self-reporting biases. During the data collection, we ensured confidentiality for participants and provided clear instructions. However, these strategies cannot guarantee that such biases were fully minimized.

## Conclusions

This large-scale population-based cross-sectional study sheds new light on the association between airline crew members’ psychological well-being and their dietary rhythmicity. Specifically, we found that meal timing (early and late breakfast [before 8 am and after 9 am] as well as late dinner [after 8 pm]) was associated with depression and anxiety regardless of work shift. Moreover, eating window (over 12 hours) may play a role in worse mental health. Finally, meal jet lag, particularly delayed dinner, was associated with depression and anxiety and thus could be detrimental to mental health. These findings underscore the need for interventions and supportive policies that help mitigate the adverse implications of shift work and irregular working hours for mental health. Such interventions may promote the overall well-being of airline employees and benefit the broader society given that a healthy airline industry workforce is essential to the safety of billions of air travelers worldwide.

## Notes

Supplement 1.
eMethods.
**eFigure 1.** Association Between Meal Timing and Depression Among Male and Female Participants **eFigure 2.** Association Between Meal Timing and Anxiety Among Male and Female Participants **eFigure 3.** Association Between Eating Window Time and Depression Among Male and Female Participants **eFigure 4.** Association Between Eating Window Time and Anxiety Among Male and Female Participants **eFigure 5.** Association Between Meal Jet Lag and Depression Among Male and Female Participants **eFigure 6.** Association Between Meal Jet Lag and Anxiety Among Male and Female Participants **eFigure 7.** Association Between Eating Jet Lag and Depression Among Male and Female Participants **eFigure 8.** Association Between Eating Jet Lag and Anxiety Among Male and Female Participants
eReferences

Supplement 2.
Data Sharing Statement
