# Supplementary material for: Dietary Rhythmicity and Mental Health Among Airline Personnel
Source: JAMA Netw Open. 2024 Jul 15;7(7):e2422266. doi: 10.1001/jamanetworkopen.2024.22266 (PMC12312486; doi:10.1001/jamanetworkopen.2024.22266)
Supplement: Supplement 1. — eMethods. eFigure 1. Association Between Meal Timing and Depression Among Male and Female Participants eFigure 2. Association Between Meal Timing and Anxiety Among Male and Female Participants eFigure 3. Association Between Eating Window Time and Depression Among Male and Female Participants eFigure 4. Association Between Eating Window Time and Anxiety Among Male and Female Participants eFigure 5. Association Between Meal Jet Lag and Depression Among Male and Female Participants eFigure 6. Association Between Meal Jet Lag and Anxiety Among Male and Female Participants eFigure 7. Association Between Eating Jet Lag and Depression Among Male and Female Participants eFigure 8. Association Between Eating Jet Lag and Anxiety Among Male and Female Participants eReferences [file jamanetwopen-e2422266-s001.pdf]

Supplemental Online Content

Zhang E, Li H, Han H, et al. Dietary rhythmicity and mental health among airline personnel. *JAMA Netw Open*. 2024;7(7):e2422266. doi:10.1001/jamanetworkopen.2024.22266

eMethods
eFigure 1. Association Between Meal Timing and Depression Among Male and Female Participants
eFigure 2. Association Between Meal Timing and Anxiety Among Male and Female Participants
eFigure 3. Association Between Eating Window Time and Depression Among Male and Female Participants
eFigure 4. Association Between Eating Window Time and Anxiety Among Male and Female Participants
eFigure 5. Association Between Meal Jet Lag and Depression Among Male and Female Participants
eFigure 6. Association Between Meal Jet Lag and Anxiety Among Male and Female Participants
eFigure 7. Association Between Eating Jet Lag and Depression Among Male and Female Participants
eFigure 8. Association Between Eating Jet Lag and Anxiety Among Male and Female Participants
eReferences

This supplemental material has been provided by the authors to give readers additional information about their work.

eMethods

Detailed description of the Civil Aviation Health Cohort of China (CAHCC)

This study used data from the CAHCC, an ongoing large-scale health surveillance cohort aiming to promote physical and mental health in pilots, flight attendants, and aviation security staff in China. The sample for the CAHCC initially consisted of aircrews from 10 airlines, including those from cargo and passenger carriers with domestic and international routes were included. The attended airlines were at varying capacities, from large-capacity airlines with thousands of aircrews to small-capacity airlines with hundreds of aircrews. As the CAHCC grows, the continuous addition of new airlines provides constant infusions of fresh blood into the representation data. As of article submission, more than 30,000 aircrew members from 12 major airlines have consented to participate in the cohort, geographically covering most provincial administrative regions in China. Prior to the first wave of CAHCC's survey, comprehensive interviews were conducted with aircrew representatives from all collaborating airlines to understand their subjective physical and mental health complaints and needs. The interviewers expressed a strong willingness to participate and provide detailed information regarding the subjective physical and mental health conditions of airline staff. Our research team recorded a video introduction of the detailed purpose and significance of this cohort before conducting the research. All aircrew members were encouraged to view the introduction and explanation before participation. Since aircrew members are constantly traveling, a web-based online survey was generated on Wenjuanxing survey platform (https://www.wjx.cn/) that can be filled out despite the physical location. In the first survey wave, an access link to the survey was sent to 24760 airline employees ages 18 and 60 via text messages from December 2022 to March 2023. The link also contains a full explanation of the research and electronic consent. Only participants who read and signed the electronic informed consent were able to access the questionnaire. For each airline company, our team created a private online chat group via WeChat (Shenzhen Tencent Technology Company), which included representatives of pilots, flight attendants, and safety officers. The online chat group received daily feedback regarding the completion of the questionnaire. A total of 22,617 crew members completed the first survey, with an overall participation rate of 91.3%.

Covariates

We examined several variables as potential confounders. Sex (male and female) and total flight participation hours were reported. Age was calculated based on their reported year and month of birth. BMI was calculated from self-reported height and weight using the following formula: BMI = weight (kg) / height (m^2), and participants were categorized as underweight (<18.5), normal (18.5-23.9), and overweight or obese (≥24.0) based on BMI cutoffs for Chinese adults[1]. Participants also reported their education level (junior college or below, bachelor's degree, and master's degree or above) and current marital status (single, stable partner, married, and divorced or widowhood), smoking status (never, former, or current), alcohol use day per month (0, 1, or ≥2) and self-reported annual personal income was categorized into the following three categories: < 50000 Chinese Yuan, 50000-150000 Chinese Yuan and >150000 Chinese Yuan (CNY:USD currency was approximately 1:7). Physical activity was accessed by the Chinese version of the International Physical Activity Questionnaire, a standardized questionnaire[2], which has been well-validated in Chinese adults[3]. Participants were categorized into two groups (inactive or active) based on whether they had 150 minutes of moderate to vigorous physical activity in the past 7 days[4]. The self-reported sleep duration were categorized into two groups (<7 h/d and ≥7 h/d) since sleep duration ranging from 7 to 9 hours was considered to be normal.[5] Social jet lag, defined as the difference in sleep midpoint times between workdays and rest days, was calculated using participants' self-reported sleep and wake-up time points on workdays and rest days, and categorized into two categories (<1 h or ≥1 h) based on previous research.[6]

© 2024 Zhang E et al. *JAMA Network Open*

eFigure 1. Association between meal timing and depression among male and female participants.

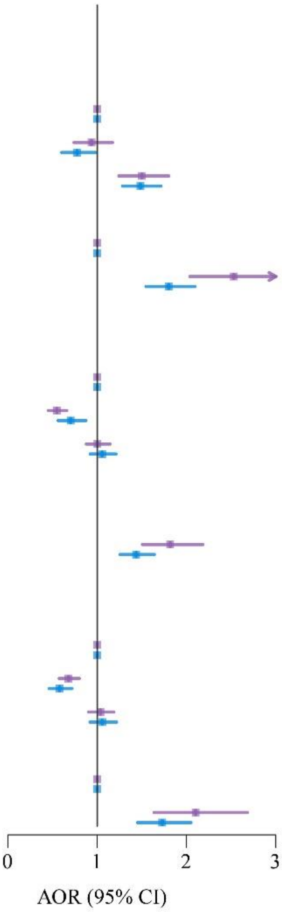

Variable	Male AOR (95% CI)	Corrected *P* value	Female AOR (95% CI)	Corrected *P* value
**Morning shift days**				
**Time of breakfast**				
Between 8AM and 9AM	1 [Refference]		1 [Refference]	
Before 8AM	0.94 [0.74–1.17]	.64	0.78 [0.60–0.99]	.06
After 9AM	1.50 [1.24–1.80]	<.001	1.48 [1.28–1.71]	<.001
**Time of dinner**				
Before and 8PM	1 [Refference]		1 [Refference]	
After 8PM	2.53 [2.04–3.13]	<.001	1.80 [1.55–2.09]	<.001
**Night shift days**				
**Time of breakfast**				
Between 8AM and 9AM	1 [Refference]		1 [Refference]	
Before 8AM	0.55 [0.45–0.66]	<.001	0.70 [0.56–0.87]	.003
After 9AM	1.00 [0.88–1.14]	.97	1.06 [0.92–1.21]	.53
**Time of dinner**				
Before and 8PM	1 [Refference]		1 [Refference]	
After 8PM	1.82 [1.51–2.18]	<.001	1.44 [1.26–1.64]	<.001
**Rest days**				
**Time of breakfast**				
Between 8AM and 9AM	1 [Refference]		1 [Refference]	
Before 8AM	0.68 [0.58–0.80]	<.001	0.58 [0.46–0.72]	<.001
After 9AM	1.04 [0.90–1.19]	.64	1.06 [0.92–1.22]	.53
**Time of dinner**				
Before and 8PM	1 [Refference]		1 [Refference]	
After 8PM	2.10 [1.64–2.68]	<.001	1.73 [1.45–2.05]	<.001

The adjusted odds ratios (AORs) were adjusted for age, education level, marital status, weight status, occupation, personal annual income, total flight participation hours, the ratio of the number of morning shifts to the number of night shifts per week, sleep duration, social jet lag, physical activity level, tobacco use, and alcohol use. All the P values are presented after Benjamini-Hochberg multiple testing adjustments.

© 2024 Zhang E et al. *JAMA Network Open*

eFigure 2. Association between meal timing and anxiety among male and female participants.

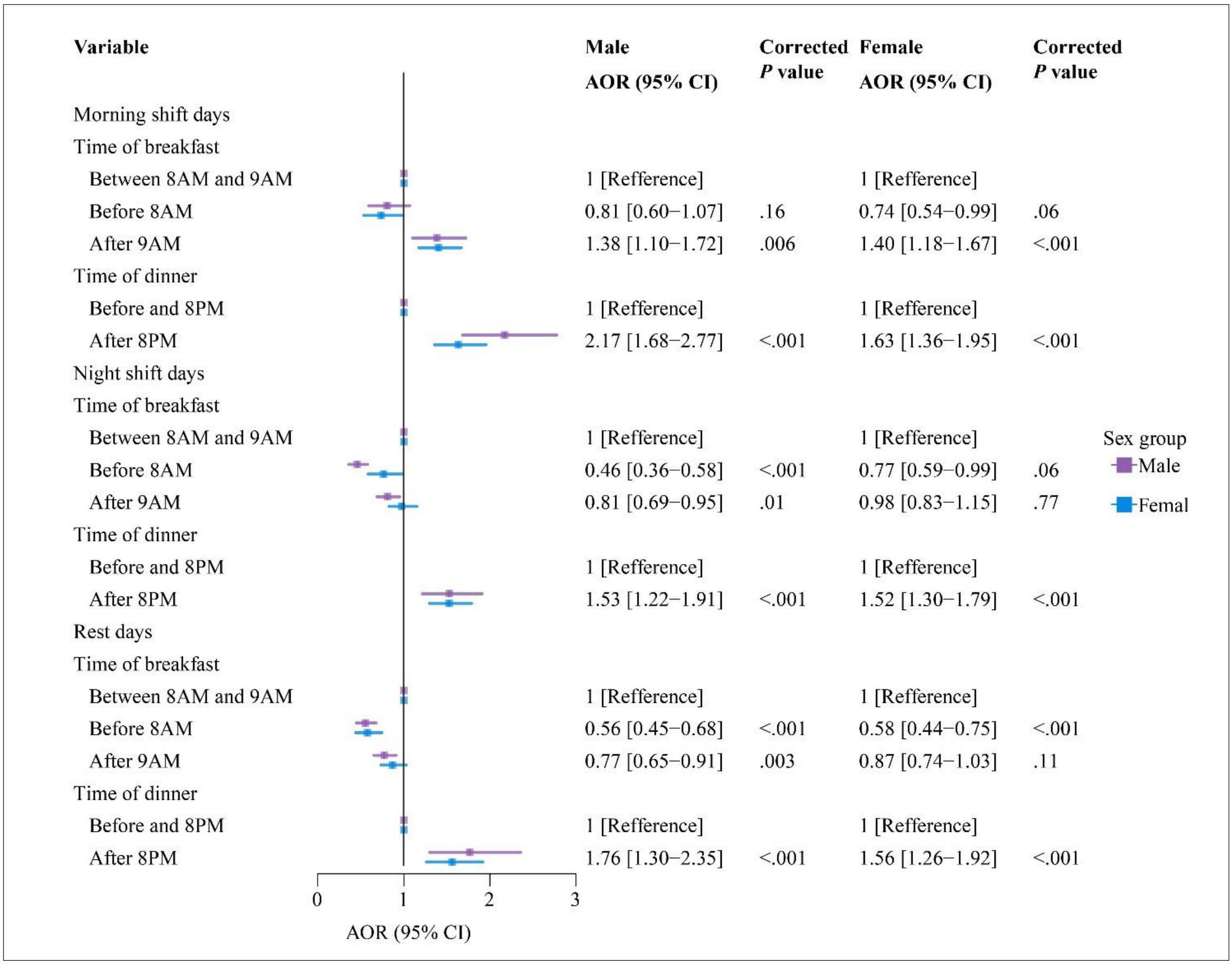

The adjusted odds ratios (AORs) were adjusted for age, education level, marital status, weight status, occupation, personal annual income, total flight participation hours, the ratio of the number of morning shifts to the number of night shifts per week, sleep duration, social jet lag, physical activity level, tobacco use, and alcohol use. All the P values are presented after Benjamini-Hochberg multiple testing adjustments.

© 2024 Zhang E et al. *JAMA Network Open*

eFigure 3. Association between eating window time and depression among male and female participants

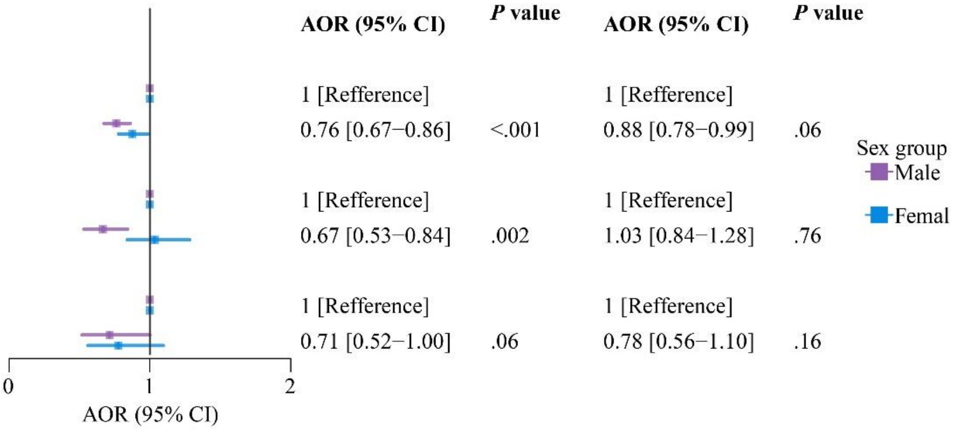

Variable	Male AOR (95% CI)	Corrected *P* value	Female AOR (95% CI)	Corrected *P* value
**Morning shift days**				
> 12 hours	1 [Refference]		1 [Refference]	
≤ 12 hours	0.76 [0.67−0.86]	<.001	0.88 [0.78−0.99]	.06
**Night shift days**				
> 12 hours	1 [Refference]		1 [Refference]	
≤ 12 hours	0.67 [0.53−0.84]	.002	1.03 [0.84−1.28]	.76
**Rest days**				
> 12 hours	1 [Refference]		1 [Refference]	
≤ 12 hours	0.71 [0.52−1.00]	.06	0.78 [0.56−1.10]	.16

The adjusted odds ratios (AORs) were adjusted for age, education level, marital status, weight status, occupation, personal annual income, total flight participation hours, the ratio of the number of morning shifts to the number of night shifts per week, sleep duration, social jet lag, physical activity level, tobacco use, and alcohol use. All the P values are presented after Benjamini-Hochberg multiple testing adjustments.

eFigure 4. Association between eating window time and anxiety among male and female participants

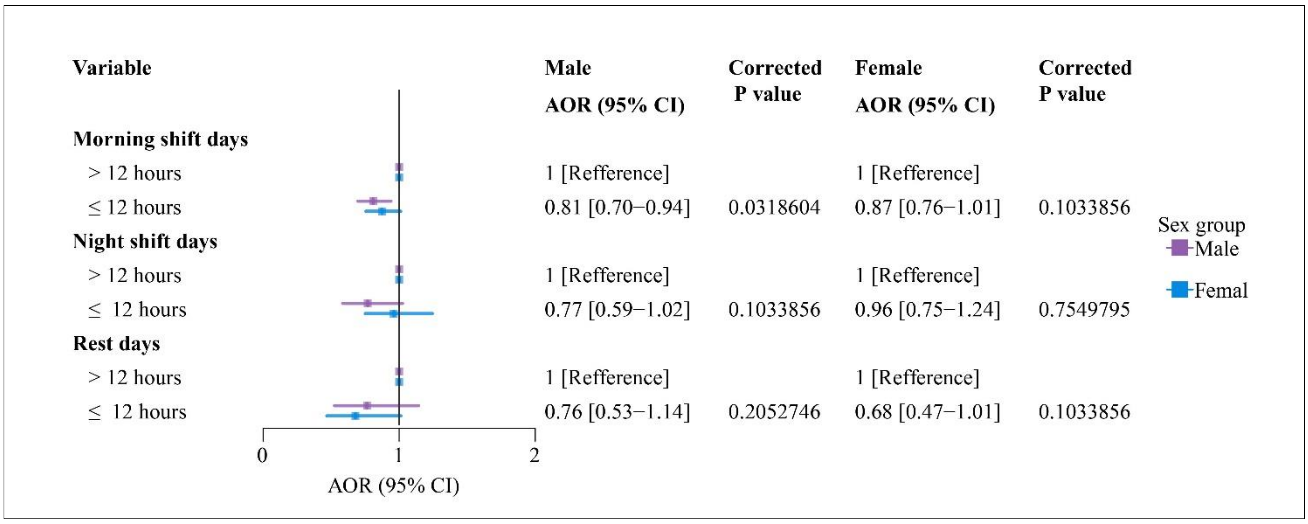

The adjusted odds ratios (AORs) were adjusted for age, education level, marital status, weight status, occupation, personal annual income, total flight participation hours, the ratio of the number of morning shifts to the number of night shifts per week, sleep duration, social jet lag, physical activity level, tobacco use, and alcohol use. All the P values are presented after Benjamini-Hochberg multiple testing adjustments.

© 2024 Zhang E et al. *JAMA Network Open*

eFigure 5. Association between meal jet lag and depression among male and female participants.

Variable	Male AOR (95% CI)	Corrected *P* value	Female AOR (95% CI)	Corrected *P* value
**Morning shift days**				
**Breakfast jet lag**				
Maintenance	1 [Refference]		1 [Refference]	
Advance	0.96 [0.85−1.09]	.63	0.92 [0.81−1.04]	.35
Delay	1.21 [0.92−1.56]	.35	1.12 [0.85−1.46]	.54
**Dinner jet lag**				
Maintenance	1 [Refference]		1 [Refference]	
Advance	1.12 [0.89−1.41]	.48	1.10 [0.91−1.32]	.48
Delay	1.64 [1.33−2.00]	<.001	1.25 [1.06−1.48]	.04
**Night shift days**				
**Breakfast jet lag**				
Maintenance	1 [Refference]		1 [Refference]	
Advance	0.94 [0.77−1.14]	.63	0.97 [0.80−1.17]	.77
Delay	0.78 [0.62−0.97]	.10	0.96 [0.76−1.19]	.74
**Dinner jet lag**				
Maintenance	1 [Refference]		1 [Refference]	
Advance	1.36 [1.02−1.80]	.10	1.22 [0.96−1.53]	.27
Delay	1.45 [1.20−1.74]	<.001	1.09 [0.94−1.25]	.47

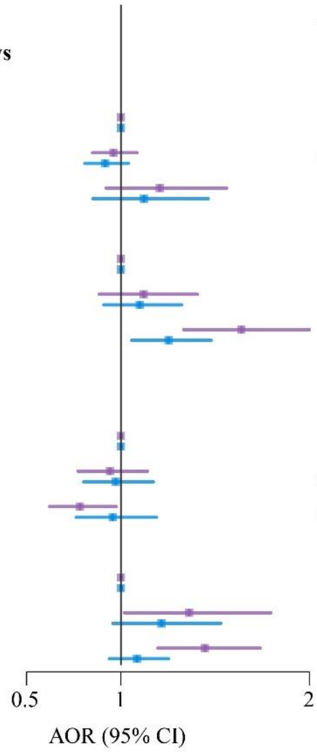

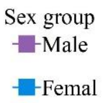

The adjusted odds ratios (AORs) were adjusted for age, education level, marital status, weight status, occupation, personal annual income, total flight participation hours, the ratio of the number of morning shifts to the number of night shifts per week, sleep duration, social jet lag, physical activity level, tobacco use, and alcohol use. All the P values are presented after Benjamini-Hochberg multiple testing adjustments.

© 2024 Zhang E et al. *JAMA Network Open*

eFigure 6. Association between meal jet lag and anxiety among male and female participants.

Variable	Male AOR (95% CI)	Corrected *P* value	Female AOR (95% CI)	Corrected *P* value
**Morning shift days**				
**Breakfast jet lag**				
Maintenance	1 [Refference]		1 [Refference]	
Advance	0.76 [0.65−0.88]	.004	0.82 [0.71−0.96]	.05
Delay	0.99 [0.72−1.35]	>.99	1.12 [0.82−1.53]	.64
**Dinner jet lag**				
Maintenance	1 [Refference]		1 [Refference]	
Advance	0.77 [0.55−1.04]	.19	1.14 [0.90−1.43]	.46
Delay	1.37 [1.07−1.74]	.05	1.32 [1.08−1.61]	.05
**Night shift days**				
**Breakfast jet lag**				
Maintenance	1 [Refference]		1 [Refference]	
Advance	0.89 [0.69−1.12]	.52	1.00 [0.79−1.25]	>.99
Delay	0.91 [0.70−1.17]	.64	1.24 [0.96−1.58]	.19
**Dinner jet lag**				
Maintenance	1 [Refference]		1 [Refference]	
Advance	1.07 [0.73−1.51]	.82	1.08 [0.79−1.44]	.77
Delay	1.28 [1.02−1.60]	.10	1.17 [0.98−1.39]	.19

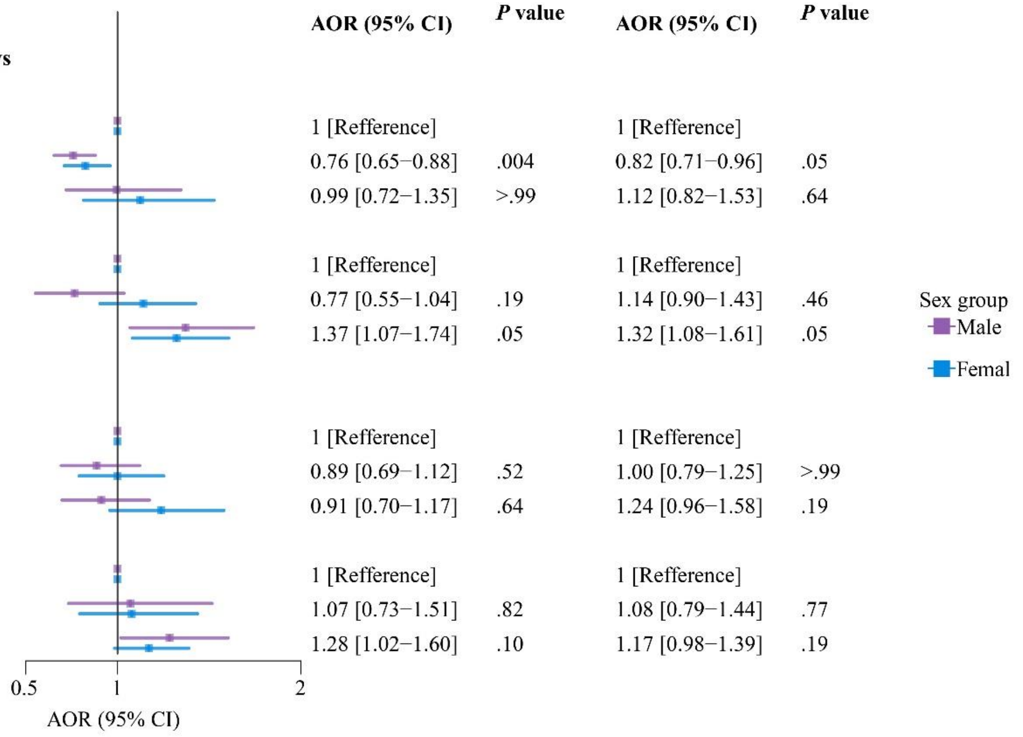

The adjusted odds ratios (AORs) were adjusted for age, education level, marital status, weight status, occupation, personal annual income, total flight participation hours, the ratio of the number of morning shifts to the number of night shifts per week, sleep duration, social jet lag, physical activity level, tobacco use, and alcohol use. All the P values are presented after Benjamini-Hochberg multiple testing adjustments.

eFigure 7. Association between eating jet lag and depression among male and female participants.

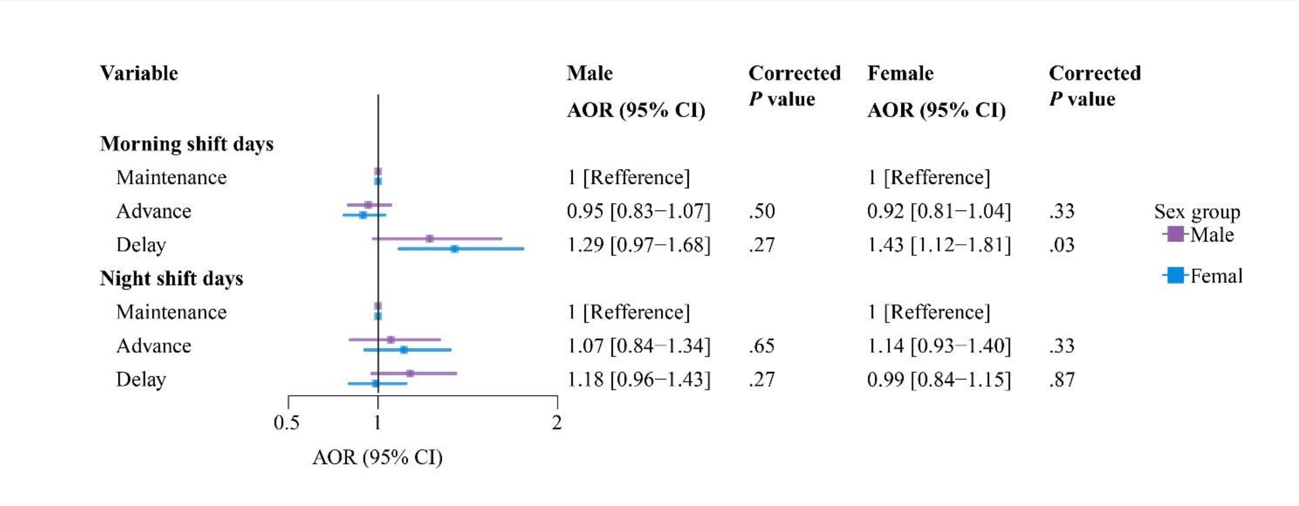

The adjusted odds ratios (AORs) were adjusted for age, education level, marital status, weight status, occupation, personal annual income, total flight participation hours, the ratio of the number of morning shifts to the number of night shifts per week, sleep duration, social jet lag, physical activity level, tobacco use, and alcohol use. All the P values are presented after Benjamini-Hochberg multiple testing adjustments.

© 2024 Zhang E et al. *JAMA Network Open*

eFigure 8. Association between eating jet lag and anxiety among male and female participants.

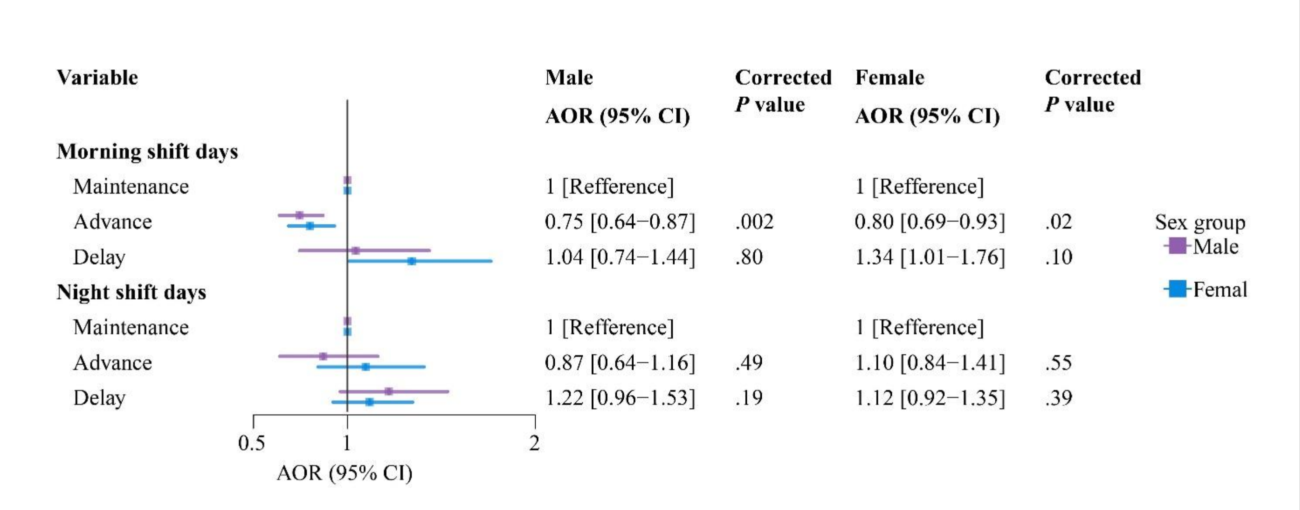

The adjusted odds ratios (AORs) were adjusted for age, education level, marital status, weight status, occupation, personal annual income, total flight participation hours, the ratio of the number of morning shifts to the number of night shifts per week, sleep duration, social jet lag, physical activity level, tobacco use, and alcohol use. All the P values are presented after Benjamini-Hochberg multiple testing adjustments.

© 2024 Zhang E et al. *JAMA Network Open*

eReferences

1. National Health Commission of the People's Republic of China. Criteria of weight for adults. Published online August 8, 2013. Accessed December 11, 2023. http://www.nhc.gov.cn/wjw/yingyang/201308/a233d450fdbc47c5ad4f08b7e394d1e8.shtml

2. Craig CL, Marshall AL, Sjöström M, et al. International physical activity questionnaire: 12-country reliability and validity. *Med Sci Sports Exerc*. 2003;35(8):1381-1395. doi:10.1249/01.MSS.0000078924.61453.FB

3. Macfarlane D, Chan A, Cerin E. Examining the validity and reliability of the Chinese version of the International Physical Activity Questionnaire, long form (IPAQ-LC). *Public Health Nutr*. 2011;14(3):443-450. doi:10.1017/S1368980010002806

4. Bull FC, Al-Ansari SS, Biddle S, et al. World Health Organization 2020 guidelines on physical activity and sedentary behaviour. *Br J Sports Med*. 2020;54(24):1451-1462. doi:10.1136/bjsports-2020-102955

5. Hirshkowitz M, Whiton K, Albert SM, et al. National Sleep Foundation's sleep time duration recommendations: methodology and results summary. *Sleep Health*. 2015;1(1):40-43. doi:10.1016/j.sleh.2014.12.010

6. Min J, Jang TW, Lee HE, Cho SS, Kang MY. Social jetlag and risk of depression: Results from the Korea National Health and Nutrition Examination Survey. *J Affect Disord*. 2023;323:562-569. doi:10.1016/j.jad.2022.12.010

© 2024 Zhang E et al. *JAMA Network Open*
