# Supplementary material for: Dietary Rhythmicity and Mental Health Among Airline Personnel
Source: JAMA Netw Open. 2024 Jul 15;7(7):e2422266. doi: 10.1001/jamanetworkopen.2024.22266 (PMC12312486; doi:10.1001/jamanetworkopen.2024.22266)
Supplement: Supplement 2. — Data Sharing Statement [file jamanetwopen-e2422266-s002.pdf]

Data Sharing Statement

Zhang. Dietary Rhythmicity and Mental Health Among Airline Personnel. *JAMA Netw Open*. Published July 15, 2024. doi:10.1001/jamanetworkopen.2024.22266

Data
Data available: No
